# 3a,11b-Dihydr­oxy-2-oxo-2,3,3a,11b-tetra­hydro-1*H*-imidazo[4,5-*f*][1,10]phenanthrolin-7-ium chloride

**DOI:** 10.1107/S160053680802093X

**Published:** 2008-07-16

**Authors:** Ying Huang, Ming-Hua Chen, Yun-Qian Zhang, Sai-Feng Xue, Zhu Tao

**Affiliations:** a Key Laboratory of Macrocyclic and Supramolecular Chemistry of Guizhou Province, Guizhou University, Guiyang 550025, People’s Republic of China; b Institute of Applied Chemistry, Guizhou University, Guiyang 550025, People’s Republic of China

## Abstract

In the crystal structure of the title compound, C_13_H_11_N_4_O_3_^+^·Cl^−^, the dihedral angle between the two pyridine rings is 9.72 (9) Å. Ions are linked *via* N—H⋯Cl, O—H⋯Cl and O—H⋯O hydrogen bonds, forming a three-dimensional framework.

## Related literature

For general background, see: Zhao *et al.* (2004 ▶); Zheng *et al.* (2005 ▶).
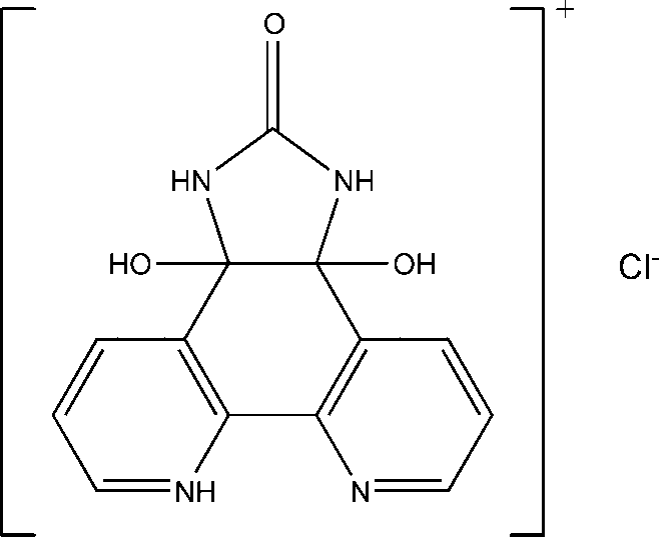


## Experimental

### 

#### Crystal data

C_13_H_11_N_4_O_3_^+^·Cl^−^
*M* *_r_* = 306.71
Monoclinic, 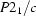

*a* = 7.9420 (13) Å
*b* = 20.352 (3) Å
*c* = 8.2972 (14) Å
β = 106.620 (5)°
*V* = 1285.1 (4) Å^3^
*Z* = 4
Mo *K*α radiation
μ = 0.31 mm^−1^
*T* = 293 (2) K
0.31 × 0.22 × 0.19 mm

#### Data collection

Bruker APEXII CCD area-detector diffractometer
Absorption correction: multi-scan (*SADABS*; Bruker, 2005 ▶) *T*_min_ = 0.909, *T*_max_ = 0.943
13480 measured reflections
2261 independent reflections
2094 reflections with *I* > 2σ(*I*)
*R*_int_ = 0.022

#### Refinement

*R*[*F* ^2^ > 2σ(*F* ^2^)] = 0.037
*wR*(*F* ^2^) = 0.103
*S* = 1.05
2261 reflections
190 parameters
H-atom parameters constrained
Δρ_max_ = 0.48 e Å^−3^
Δρ_min_ = −0.52 e Å^−3^

### 

Data collection: *APEX2* (Bruker, 2005 ▶); cell refinement: *SAINT* (Bruker, 2005 ▶); data reduction: *SAINT*; program(s) used to solve structure: *SHELXS97* (Sheldrick, 2008 ▶); program(s) used to refine structure: *SHELXL97* (Sheldrick, 2008 ▶); molecular graphics: *ORTEP-3 for Windows* (Farrugia, 1997 ▶); software used to prepare material for publication: *WinGX* (Farrugia, 1999 ▶).

## Supplementary Material

Crystal structure: contains datablocks global, I. DOI: 10.1107/S160053680802093X/at2573sup1.cif

Structure factors: contains datablocks I. DOI: 10.1107/S160053680802093X/at2573Isup2.hkl

Additional supplementary materials:  crystallographic information; 3D view; checkCIF report

## Figures and Tables

**Table 1:** Hydrogen-bond geometry (Å, °)

*D*—H⋯*A*	*D*—H	H⋯*A*	*D*⋯*A*	*D*—H⋯*A*
N1—H1*A*⋯Cl1^i^	0.86	2.41	3.1512 (17)	145
N3—H3*A*⋯O2^ii^	0.86	2.65	3.146 (2)	118
N4—H4⋯Cl1^iii^	0.86	2.50	3.2490 (16)	147
O2—H2*A*⋯Cl1	0.82	2.28	3.0712 (15)	163
O3—H3*B*⋯O1^iv^	0.82	1.89	2.6867 (18)	165

Symmetry codes: (i) 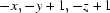; (ii) 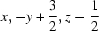; (iii) 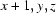; (iv) 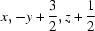.

**Fig. 1.**
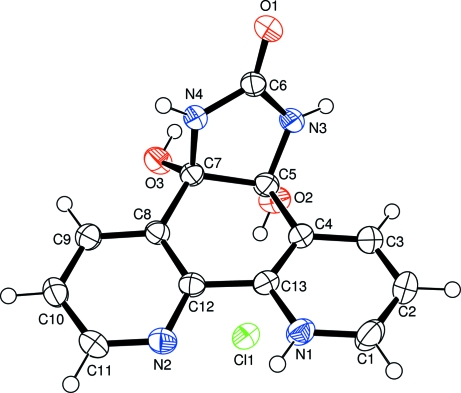
The molecular structure of (I) showing the atom-labelling scheme. Displacement ellipsoids are drawn at the 50% probability level.

## supplementary crystallographic information

### Comment

Recent year, we used different alkyl-substituted glycolurils as the building blocks to synthesize the partially alkyl substituted cucurbit[*n*]urils (Zhao *et al.*, 2004; Zheng *et al.*, 2005). In this work, we further report the crystal structure of a phenanthroline-substituted semi-glycoluril.

In the title compound (I), (Fig. 1), consists of organic cations, Cl^-^ anions. The dihedral angle between two pyridine rings is 9.72 (9) Å. Molecules are linked *via* N—H···Cl, O—H···Cl and O—H···O hydrogen bonds forming a three-dimensional framework. (Table 1).

### Experimental

1,10-Phenanthroline-5,6-dione (3.00 g,14.29 mmol) and carbamide (15.00 g, 250 mmol) were dissolved in acetic acid glacial (120 mL) and hydrochloric acid (5 mL) at room temperature. There was a lot of deposit after the mixture were stirred 5 h. Filtrate, solid was washed by ethanol, drying, gained white powder 2.46 g [yield: 63%].

### Refinement

H atoms were placed in calculated positions with C—H = 0.93, N—H = 0.86 and O—H = 0.82 Å and refined as riding, with *U*_iso_(H) = 1.2-1.5*U*_eq_.

### Crystal data

**Table:**

C_13_H_11_N_4_O_3_^+^·Cl^–^	*F*_000_ = 632
*M**_r_* = 306.71	*D*_x_ = 1.585 Mg m^−^^3^
Monoclinic, *P*2_1_/*c*	Mo *K*α radiation λ = 0.71073 Å
Hall symbol: -P 2ybc	Cell parameters from 2261 reflections
*a* = 7.9420 (13) Å	θ = 2.0–25.0º
*b* = 20.352 (3) Å	µ = 0.31 mm^−^^1^
*c* = 8.2972 (14) Å	*T* = 293 (2) K
β = 106.620 (5)º	Prism, colourless
*V* = 1285.1 (4) Å^3^	0.31 × 0.22 × 0.19 mm
*Z* = 4	

### Data collection

**Table:**

Bruker APEXII CCD area-detector diffractometer	2261 independent reflections
Radiation source: fine-focus sealed tube	2094 reflections with *I* > 2σ(*I*)
Monochromator: graphite	*R*_int_ = 0.022
*T* = 293(2) K	θ_max_ = 25.0º
φ and ω scans	θ_min_ = 2.0º
Absorption correction: multi-scan(SADABS; Bruker, 2005)	*h* = −8→9
*T*_min_ = 0.909, *T*_max_ = 0.943	*k* = −24→24
13480 measured reflections	*l* = −9→8

### Refinement

**Table:**

Refinement on *F*^2^	Secondary atom site location: difference Fourier map
Least-squares matrix: full	Hydrogen site location: inferred from neighbouring sites
*R*[*F*^2^ > 2σ(*F*^2^)] = 0.037	H-atom parameters constrained
*wR*(*F*^2^) = 0.103	*w* = 1/[σ^2^(*F*_o_^2^) + (0.0589*P*)^2^ + 0.7326*P*] where *P* = (*F*_o_^2^ + 2*F*_c_^2^)/3
*S* = 1.05	(Δ/σ)_max_ = 0.001
2261 reflections	Δρ_max_ = 0.48 e Å^−^^3^
190 parameters	Δρ_min_ = −0.51 e Å^−^^3^
Primary atom site location: structure-invariant direct methods	Extinction correction: none

### Special details

**Table:**

Geometry. All e.s.d.'s (except the e.s.d. in the dihedral angle between two l.s. planes) are estimated using the full covariance matrix. The cell e.s.d.'s are taken into account individually in the estimation of e.s.d.'s in distances, angles and torsion angles; correlations between e.s.d.'s in cell parameters are only used when they are defined by crystal symmetry. An approximate (isotropic) treatment of cell e.s.d.'s is used for estimating e.s.d.'s involving l.s. planes.
Refinement. Refinement of *F*^2^ against ALL reflections. The weighted *R*-factor *wR* and goodness of fit *S* are based on *F*^2^, conventional *R*-factors *R* are based on *F*, with *F* set to zero for negative *F*^2^. The threshold expression of *F*^2^ > σ(*F*^2^) is used only for calculating *R*-factors(gt) *etc*. and is not relevant to the choice of reflections for refinement. *R*-factors based on *F*^2^ are statistically about twice as large as those based on *F*, and *R*- factors based on ALL data will be even larger.

### Fractional atomic coordinates and isotropic or equivalent isotropic displacement parameters (Å^2^)

**Table:**

	*x*	*y*	*z*	*U*_iso_*/*U*_eq_	
C1	−0.0113 (3)	0.58542 (11)	0.2547 (3)	0.0409 (5)	
H1	−0.0891	0.5658	0.1616	0.049*	
C2	−0.0166 (3)	0.65219 (11)	0.2779 (3)	0.0446 (5)	
H2	−0.0982	0.6781	0.2015	0.054*	
C3	0.1010 (2)	0.68003 (10)	0.4159 (3)	0.0376 (5)	
H3	0.0960	0.7249	0.4352	0.045*	
C4	0.2275 (2)	0.64158 (9)	0.5271 (2)	0.0280 (4)	
C5	0.3591 (2)	0.67304 (8)	0.6779 (2)	0.0288 (4)	
C6	0.6083 (2)	0.70941 (8)	0.6106 (2)	0.0286 (4)	
C7	0.5217 (2)	0.62729 (8)	0.7653 (2)	0.0265 (4)	
C8	0.4878 (2)	0.55393 (8)	0.7408 (2)	0.0250 (4)	
C9	0.6001 (2)	0.50849 (9)	0.8421 (2)	0.0311 (4)	
H9	0.6963	0.5226	0.9282	0.037*	
C10	0.5678 (2)	0.44209 (9)	0.8141 (2)	0.0325 (4)	
H10	0.6423	0.4111	0.8805	0.039*	
C11	0.4231 (3)	0.42256 (9)	0.6860 (2)	0.0327 (4)	
H11	0.4009	0.3778	0.6693	0.039*	
C12	0.3479 (2)	0.52914 (8)	0.6134 (2)	0.0258 (4)	
C13	0.2261 (2)	0.57459 (8)	0.5007 (2)	0.0261 (4)	
N1	0.10578 (19)	0.54886 (8)	0.36612 (19)	0.0314 (4)	
H1A	0.1046	0.5070	0.3516	0.038*	
N2	0.3139 (2)	0.46487 (7)	0.58529 (19)	0.0315 (4)	
N3	0.44790 (19)	0.72802 (7)	0.6258 (2)	0.0337 (4)	
H3A	0.4054	0.7671	0.6071	0.040*	
N4	0.64752 (19)	0.64940 (7)	0.68046 (18)	0.0284 (3)	
H4	0.7373	0.6268	0.6752	0.034*	
O1	0.70045 (17)	0.74179 (6)	0.54285 (17)	0.0374 (3)	
O2	0.26989 (17)	0.69570 (7)	0.79061 (17)	0.0389 (3)	
H2A	0.2216	0.6647	0.8224	0.058*	
O3	0.58135 (18)	0.63742 (6)	0.93855 (15)	0.0355 (3)	
H3B	0.6013	0.6766	0.9577	0.053*	
Cl1	0.03122 (6)	0.58193 (2)	0.83116 (6)	0.03931 (18)	

### Atomic displacement parameters (Å^2^)

**Table:**

	*U*^11^	*U*^22^	*U*^33^	*U*^12^	*U*^13^	*U*^23^
C1	0.0294 (10)	0.0529 (13)	0.0332 (10)	−0.0040 (9)	−0.0026 (8)	0.0010 (9)
C2	0.0330 (10)	0.0466 (12)	0.0450 (12)	0.0029 (9)	−0.0037 (9)	0.0121 (10)
C3	0.0304 (10)	0.0314 (10)	0.0473 (12)	0.0010 (8)	0.0053 (8)	0.0062 (8)
C4	0.0242 (9)	0.0289 (9)	0.0305 (9)	−0.0007 (7)	0.0073 (7)	0.0014 (7)
C5	0.0289 (9)	0.0228 (8)	0.0336 (10)	−0.0010 (7)	0.0071 (7)	−0.0031 (7)
C6	0.0302 (9)	0.0226 (8)	0.0285 (9)	−0.0043 (7)	0.0014 (7)	−0.0017 (7)
C7	0.0274 (9)	0.0252 (9)	0.0239 (8)	−0.0032 (7)	0.0028 (7)	−0.0008 (7)
C8	0.0270 (8)	0.0242 (9)	0.0239 (8)	−0.0019 (7)	0.0074 (7)	0.0005 (6)
C9	0.0308 (9)	0.0313 (9)	0.0282 (9)	−0.0021 (7)	0.0037 (7)	0.0017 (7)
C10	0.0357 (10)	0.0285 (9)	0.0331 (10)	0.0041 (8)	0.0099 (8)	0.0067 (8)
C11	0.0402 (10)	0.0225 (9)	0.0369 (10)	−0.0016 (7)	0.0133 (8)	0.0001 (7)
C12	0.0266 (8)	0.0247 (8)	0.0270 (9)	−0.0017 (7)	0.0092 (7)	−0.0021 (7)
C13	0.0230 (8)	0.0296 (9)	0.0255 (9)	−0.0025 (7)	0.0065 (7)	−0.0014 (7)
N1	0.0281 (8)	0.0325 (8)	0.0305 (8)	−0.0031 (6)	0.0031 (6)	−0.0043 (6)
N2	0.0337 (8)	0.0258 (8)	0.0328 (8)	−0.0031 (6)	0.0061 (6)	−0.0036 (6)
N3	0.0296 (8)	0.0197 (7)	0.0488 (10)	0.0004 (6)	0.0065 (7)	0.0035 (7)
N4	0.0258 (7)	0.0233 (7)	0.0348 (8)	0.0006 (6)	0.0064 (6)	0.0037 (6)
O1	0.0358 (7)	0.0304 (7)	0.0443 (8)	−0.0045 (6)	0.0088 (6)	0.0097 (6)
O2	0.0381 (7)	0.0349 (7)	0.0468 (8)	−0.0022 (6)	0.0172 (6)	−0.0118 (6)
O3	0.0482 (8)	0.0293 (7)	0.0239 (7)	−0.0074 (6)	0.0021 (6)	−0.0027 (5)
Cl1	0.0309 (3)	0.0414 (3)	0.0425 (3)	−0.00238 (18)	0.0053 (2)	−0.0005 (2)

### Geometric parameters (Å, °)

**Table:**

C1—N1	1.335 (3)	C7—C8	1.520 (2)
C1—C2	1.375 (3)	C8—C9	1.388 (2)
C1—H1	0.9300	C8—C12	1.391 (2)
C2—C3	1.376 (3)	C9—C10	1.383 (3)
C2—H2	0.9300	C9—H9	0.9300
C3—C4	1.394 (3)	C10—C11	1.382 (3)
C3—H3	0.9300	C10—H10	0.9300
C4—C13	1.380 (2)	C11—N2	1.333 (2)
C4—C5	1.523 (2)	C11—H11	0.9300
C5—O2	1.403 (2)	C12—N2	1.342 (2)
C5—N3	1.453 (2)	C12—C13	1.466 (2)
C5—C7	1.589 (2)	C13—N1	1.350 (2)
C6—O1	1.233 (2)	N1—H1A	0.8600
C6—N4	1.350 (2)	N3—H3A	0.8600
C6—N3	1.369 (2)	N4—H4	0.8600
C7—O3	1.394 (2)	O2—H2A	0.8200
C7—N4	1.449 (2)	O3—H3B	0.8200
			
N1—C1—C2	119.82 (18)	C12—C8—C7	122.01 (15)
N1—C1—H1	120.1	C10—C9—C8	119.56 (17)
C2—C1—H1	120.1	C10—C9—H9	120.2
C1—C2—C3	118.87 (19)	C8—C9—H9	120.2
C1—C2—H2	120.6	C11—C10—C9	118.93 (17)
C3—C2—H2	120.6	C11—C10—H10	120.5
C2—C3—C4	120.59 (19)	C9—C10—H10	120.5
C2—C3—H3	119.7	N2—C11—C10	123.04 (16)
C4—C3—H3	119.7	N2—C11—H11	118.5
C13—C4—C3	118.54 (17)	C10—C11—H11	118.5
C13—C4—C5	121.21 (16)	N2—C12—C8	124.23 (16)
C3—C4—C5	120.21 (16)	N2—C12—C13	116.16 (15)
O2—C5—N3	109.01 (14)	C8—C12—C13	119.61 (15)
O2—C5—C4	109.09 (14)	N1—C13—C4	119.09 (16)
N3—C5—C4	110.92 (15)	N1—C13—C12	117.68 (15)
O2—C5—C7	112.82 (15)	C4—C13—C12	123.23 (16)
N3—C5—C7	100.76 (13)	C1—N1—C13	122.99 (17)
C4—C5—C7	113.94 (14)	C1—N1—H1A	118.5
O1—C6—N4	125.80 (17)	C13—N1—H1A	118.5
O1—C6—N3	125.70 (16)	C11—N2—C12	117.26 (16)
N4—C6—N3	108.49 (15)	C6—N3—C5	110.99 (14)
O3—C7—N4	112.13 (14)	C6—N3—H3A	124.5
O3—C7—C8	106.15 (13)	C5—N3—H3A	124.5
N4—C7—C8	111.12 (14)	C6—N4—C7	112.54 (15)
O3—C7—C5	112.10 (14)	C6—N4—H4	123.7
N4—C7—C5	100.35 (13)	C7—N4—H4	123.7
C8—C7—C5	115.14 (14)	C5—O2—H2A	109.5
C9—C8—C12	116.98 (16)	C7—O3—H3B	109.5
C9—C8—C7	120.98 (15)		
			
N1—C1—C2—C3	0.5 (3)	C9—C8—C12—N2	1.3 (3)
C1—C2—C3—C4	2.3 (3)	C7—C8—C12—N2	179.10 (16)
C2—C3—C4—C13	−3.4 (3)	C9—C8—C12—C13	−179.17 (16)
C2—C3—C4—C5	178.93 (18)	C7—C8—C12—C13	−1.4 (2)
C13—C4—C5—O2	−110.16 (18)	C3—C4—C13—N1	1.7 (3)
C3—C4—C5—O2	67.4 (2)	C5—C4—C13—N1	179.35 (15)
C13—C4—C5—N3	129.75 (17)	C3—C4—C13—C12	−177.36 (17)
C3—C4—C5—N3	−52.7 (2)	C5—C4—C13—C12	0.3 (3)
C13—C4—C5—C7	16.9 (2)	N2—C12—C13—N1	−8.7 (2)
C3—C4—C5—C7	−165.51 (16)	C8—C12—C13—N1	171.79 (15)
O2—C5—C7—O3	−21.6 (2)	N2—C12—C13—C4	170.44 (16)
N3—C5—C7—O3	94.51 (16)	C8—C12—C13—C4	−9.1 (3)
C4—C5—C7—O3	−146.68 (15)	C2—C1—N1—C13	−2.3 (3)
O2—C5—C7—N4	−140.73 (14)	C4—C13—N1—C1	1.1 (3)
N3—C5—C7—N4	−24.66 (16)	C12—C13—N1—C1	−179.75 (17)
C4—C5—C7—N4	94.16 (16)	C10—C11—N2—C12	−0.7 (3)
O2—C5—C7—C8	99.91 (17)	C8—C12—N2—C11	−0.6 (3)
N3—C5—C7—C8	−144.01 (14)	C13—C12—N2—C11	179.89 (16)
C4—C5—C7—C8	−25.2 (2)	O1—C6—N3—C5	167.40 (17)
O3—C7—C8—C9	−39.1 (2)	N4—C6—N3—C5	−12.0 (2)
N4—C7—C8—C9	83.02 (19)	O2—C5—N3—C6	142.10 (15)
C5—C7—C8—C9	−163.78 (16)	C4—C5—N3—C6	−97.76 (17)
O3—C7—C8—C12	143.18 (16)	C7—C5—N3—C6	23.22 (18)
N4—C7—C8—C12	−94.67 (19)	O1—C6—N4—C7	173.90 (17)
C5—C7—C8—C12	18.5 (2)	N3—C6—N4—C7	−6.7 (2)
C12—C8—C9—C10	−0.8 (3)	O3—C7—N4—C6	−98.97 (17)
C7—C8—C9—C10	−178.56 (16)	C8—C7—N4—C6	142.41 (15)
C8—C9—C10—C11	−0.4 (3)	C5—C7—N4—C6	20.17 (17)
C9—C10—C11—N2	1.2 (3)		

### Hydrogen-bond geometry (Å, °)

**Table:**

*D*—H···*A*	*D*—H	H···*A*	*D*···*A*	*D*—H···*A*
N1—H1A···Cl1^i^	0.86	2.41	3.1512 (17)	145
N3—H3A···O2^ii^	0.86	2.65	3.146 (2)	118
N4—H4···Cl1^iii^	0.86	2.50	3.2490 (16)	147
O2—H2A···Cl1	0.82	2.28	3.0712 (15)	163
O3—H3B···O1^iv^	0.82	1.89	2.6867 (18)	165

Symmetry codes: (i) −*x*, −*y*+1, −*z*+1; (ii) *x*, −*y*+3/2, *z*−1/2; (iii) *x*+1, *y*, *z*; (iv) *x*, −*y*+3/2, *z*+1/2.
